# Monitoring Hydroquinone Clathrates in Molecular Simulation Using Local Bond Order Parameters

**DOI:** 10.1021/acs.energyfuels.5c01091

**Published:** 2025-05-14

**Authors:** Brais R. García, Jesús Algaba, Felipe J. Blas, Martín Pérez-Rodríguez, Manuel M. Piñeiro

**Affiliations:** † Departamento de Física Aplicada, 16784 Universidade de Vigo, 36310 Vigo, Spain; ‡ Laboratorio de Simulación Molecular y Química Computacional, CIQSO-Centro de Investigación en Química Sostenible and Departamento de Ciencias Integradas, 16743 Universidad de Huelva, 21006 Huelva, Spain; § 69568 Instituto de Química Física Blas Cabrera, CSIC, E-28006 Madrid, Spain

## Abstract

Hydroquinone clathrates (HQ clathrates) are highly structured crystalline materials with promising application in carbon separation and sequestration, and also in hydrogen storage. In this study, molecular simulation techniques are employed to analyze the structure of β-HQ clathrates using local bond order parameters. The methodology is based on the definition by Steinhardt and Lechner–Dellago of the averaged bond order parameters, which allow a precise differentiation between solid and liquid phases. Using molecular dynamics simulations, we evaluate the role of guest molecules such as CO_2_ and CH_4_ in the stability and formation of clathrates. In this study, we determine and test an optimal combination of bond order parameters (*q̅*_12_–*q̅*_8_) capable of accurately characterizing phase transitions with a classification error of less than 0.001%. The proposed method is able to qualitatively and quantitatively discern the membership of each molecule to the different phases during the crystallization and dissociation processes, demonstrating its effectiveness in the study of the dynamics of HQ clathrate at different pressure and temperature conditions. The results of this work provide a solid and applicable theoretical framework intended to further provide insight into the nucleation process of this system, contributing to its understanding.

## Graphical abstract

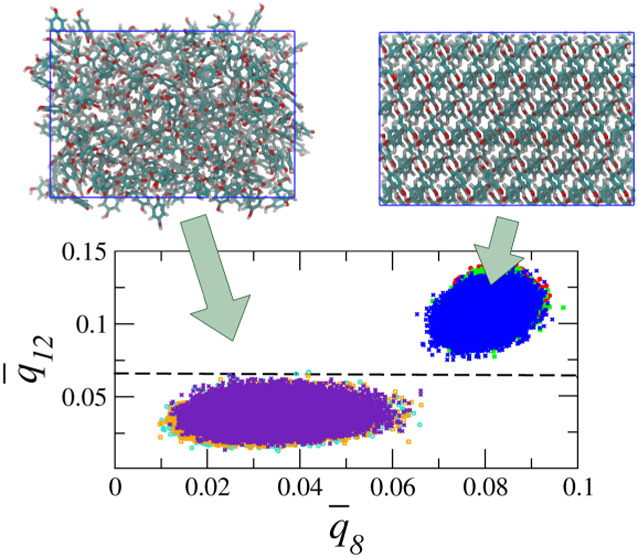

## Introduction

A clathrate is defined as a crystalline structure of nonstoichiometric composition whose ordered lattice, called a matrix, houses small atoms or molecules, known as guests. These guests are housed in spaces called cells, periodically distributed as a result of the arrangement of the constituent particles of the matrix. The ability to contain and store substances of interest has brought these substances to the attention of the scientific community and industry. It is now known that clathrates can be useful in wastewater treatment, seawater desalination, and separation of gaseous mixtures by “selective clathration”.^1^

One of the most versatile of these organic structures is the hydroquinone (HQ) clathrate.^2–4^ This clathrate has been demonstrated high selectivity for carbon dioxide (CO_2_) capture processes, crucial in atmospheric decarbonization policies, as well as in the storage of substances of interest, such as methane or hydrogen (H_2_).^5–11^ The latter is of key interest from both industrial and energetic points of view.^2,3,12,13^ This clathrate structure corresponds to the β crystalline solid phase of HQ, which has a high guest–host ratio of 1:3.^14–16^ This phase has a strong directionality due to the presence of channels arranged along one of the space dimensions. The consecutive stacking of these leads to the formation of a flexible network capable of adapting to small and large hosts without the need for a phase transition.^3,4,17^ They also provide a safe environment to store guests whose chemical stability is compromised by external environmental conditions.^2,5,18,19^

The radius of the cells arranged consecutively along the channels is estimated to be of the order of 2.5 Å.^20^ Unlike the cases of other structures of similar nature, diffusion of the individual guest particles along the linear clathrate channels is not negligible. This characteristic diffusion is a result of the consecutive transition of a guest from one cell to the next, which leads to a considerable net displacement within the structure. Other substances with similar characteristics such as hydrates, clathrates whose crystalline matrix consists entirely of water, only present diffusion within the cell in which the guest is contained.^3,4,17,21^ This fact allows β-HQ clathrates to be filled and emptied without the need for melting and recrystallization cycles.

Within the β HQ clathrate phase, it is possible to find three different structures according to their symmetry. These are called type I, II, and III, and vary depending on the nature of the guest occupying the matrix.^15,16^ Those species with spherical symmetry such as noble gases form type I structure, belonging to the space group *R*3̅.^7,22^ The flexibility and size of the crystal lattice allow large species such as argon (Ar) or xenon (Xe) to be accommodated, although their diffusion becomes less relevant as the atomic radius increases. Type II structure belongs to the *R*3 group, hosting molecules such as methanol (CH_3_OH) or hydrochloric acid (HCl). Finally, type III belongs to the *P*3 group, and it appears when the crystalline matrix hosts complex molecules such as acetonitrile.

In reference to the other HQ solid polymorphs, they do not have the inclusion ability of β phase. Three structures are known in addition to the one selected for this study, referred to as α, γ, and δ.^23–25^ The α phase is the native phase and has a guest-to-host ratio of 1:18. It has small cavities that may accommodate up to two guest particles per unit cell. The variety of guests that the crystalline matrix is able to accept is more limited, with molecules such as CO_2_ and H_2_ and noble gases of reduced atomic radius such as neon (Ne).^7,23,24,26^ The γ-HQ corresponds to the monoclinic phase, and can be synthesized in the laboratory by sublimation processes or rapid evaporation of HQ in ether.^14–16^ Finally, the δ phase is derived from the α phase by subjecting the matrix to high-pressure conditions.^23–25^

Given its multiple utilities, studying and understanding the behavior of this system is of remarkable interest to expand its range of practical application. However, the theoretical understanding of key phenomena such as nucleation or clathrate growth is still incomplete. In this context, there are different techniques that allow the estimation of nucleation and growth rates. Various enhanced sampling techniques have been developed to address rare-event challenges, including Forward Flux Sampling,^27^ Transition Path Sampling,^28–30^ Metadynamics,^31,32^ Lattice Mold,^33^ and Umbrella Sampling.^34,35^ Unfortunately, these simulations cannot provide nucleation rate estimates comparable to experimental results, as they require extremely high driving forces and, consequently, very low temperatures. In recent years, however, an alternative approach has been refined and expanded to estimate nucleation rates at higher temperatures: the Seeding technique,^36–40^ used in conjunction with Classical Nucleation Theory (CNT).^41–43^ This method has been employed by different researchers to determine nucleation rates for water, NaCl, and hydrates.^39,40,44–47^ The key point of Seeding is to define an order parameter to distinguish between the nucleus (stable phase) and the surrounding metastable phase.^48,49^ Unfortunately, different order parameter choices yield different nucleus sizes and, consequently, different nucleation rates.^50^ Therefore, having well-defined order parameters is crucial for accurately distinguishing between liquid-state and solid-state molecules during simulations.

The first set of order parameters aimed at distinguishing particle membership of a solid or liquid phase was proposed by Steinhardt et al.,^51^ based on the conception of Frank,^52^ who established the importance of local orientational symmetries in the characterization of the internal structure of three-dimensional solid and liquid systems. Therefore, the authors proposed to relate a set of spherical harmonics to the group of neighboring particles of a given reference molecule.^51^ However, the existence of thermal fluctuations overlaps the distributions related to the order parameters. This makes this ensemble unable to accurately distinguish between different localized crystal structures.

Seeking to increase the accuracy of the method, Dellago and Lechner^53^ proposed to average the various binding order parameters of the immediate neighbor list associated with the constituent particles of the system. This achieves a lower associated error that significantly improves the results. Since then, several authors have used the averaged local order parameters to distinguish phase membership in complex systems, estimate nucleation rates, and calculate interfacial free energies. Most of this work was carried out with the *q̅*_4_–*q̅*_6_ planar representation. However, it has been found that this combination may not be the most effective in all cases, and better results are obtained if it is optimized for each specific system. Particularly, some of us have extended the local order parameter of Lechner and Dellago to deal with carbon dioxide, methane, nitrogen, hydrogen, and tetrahydrofuran hydrates.^21,44,54–57^

In the case of HQ, the use of these parameters can be a major breakthrough. For this system, the growth or dissociation of a crystalline seed may not be easily observable within conventional simulation times. This is particularly evident when studying the behavior of these compounds in confined environments, as it is the case of nucleation in porous systems. The study of hydrate crystallization inside porous matrices is a very active research field nowadays.^58,59^ Ciocarlan et al.^60^ have demonstrated that using mesoporous silica materials to create clathrates-forming nanoconfinement in the H_2_ storage process notably lowers crystallization pressures, making the process more feasible for practical applications. This fact is of remarkable importance in the analysis of HQ clathrates in particular. Establishing a reliable technique that can adequately discern whether each molecule belongs to a stable or metastable state would represent a significant progress, which would lead to a more comprehensive study of this type of structured materials. Therefore, the aim of this work is to identify a suitable combination of order parameters that allows to properly discern the belonging of a particle to its corresponding phase. A qualitative analysis is proposed, detecting the presence of growth or dissociation of a β-HQ clathrate, and a quantitative analysis, determining the results at equilibrium to provide a value for the number of molecules belonging to a solid cluster.

The organization of this paper is as follows: In the next section, we describe the methodology used in the manuscript. After this, we present the simulation details and molecular models used. The results obtained in this work are discussed in detail in the following section. Finally, conclusions are presented in the last section of this work.

## Methods

According to the previous discussion, a common aspect in the simulation methods described for the estimation of nucleation rates and interfacial free energies is the need to use order parameters to distinguish between molecules with solid and liquid behavior. In the literature, numerous order parameters have been proposed for this purpose. The first to be formulated to differentiate particles in solid and liquid phases were introduced by Steinhardt et al.,^51^ based on the approach of local orientational symmetries in condensed phases proposed by Frank.^52^ According to this conception, local orientational symmetries are fundamental to characterize the internal structure of three-dimensional liquids and solids.

Steinhardt et al.^51^ proposed to associate a set of spherical harmonics, *Y*_*lm*_(**r**_*ij*_), with the neighbors of a given atom, thus defining the complex vector associated with a particle *i*

1 $$q_{\mathrm{lm}}\left(i\right)=\frac{1}{N_{b}\left(i\right)}\sum_{j=1}^{N_{b}\left(i\right)}Y_{\mathrm{lm}}\left(r_{\mathrm{ij}}\right)$$

In eq 1, *N*_*b*_(*i*) represents the number of nearest neighbors of the *i*th particle, *l* is an integer parameter, and *m* varies between −*l* and *l*. In order to efficiently distinguish not only between particles with solid and liquid behavior, but also between different crystal structures, Steinhardt et al.^51^ defined the so-called local bond order parameters

2 $$q_{l}\left(i\right)=\sqrt{\frac{4\pi }{2l+1}\sum_{m=-l}^{l}|q_{\mathrm{lm}}\left(i\right){|}^{2}}$$

Depending on the value of *l*, these parameters allow differentiating different crystalline symmetries. Frenkel et al.^48,61–64^ used these parameters to investigate homogeneous nucleation in various systems, finding that the values of *q*_4_ and *q*_6_ are particularly suitable for distinguishing between different solid structures. Subsequently, Desgranges and Delhommelle^65^ improved the method by using a linear combination *q̅*_4_–*q̅*_6_, which facilitated the identification of solid and liquid particles.

However, the local order parameters of Steinhardt et al.^51^ have limitations in the differentiation of local crystal structures due to the presence of the aforementioned thermal fluctuations, which can distort the distribution of the order parameters. To mitigate this effect, Lechner and Dellago^53^ proposed a modified version of the Steinhardt order parameters, based on averaging over the nearest-neighbor values. This implies averaging the complex vectors *q*_*l*_(*i*) calculated for a molecule and its neighbors, thus obtaining a new complex vector *q̅*_*l*_(*i*). This approach significantly improves the accuracy in determining the crystal structure of the system. Since then, several authors have used these averaged local order parameters together with the linear combination *q̅*_4_–*q̅*_6_ to successfully distinguish between solid and liquid molecules in nucleation studies and in the determination of solid–liquid interfacial free energies.

This strategy allows to reduce the influence of thermal fluctuations and improve the accuracy of crystal structure determination without requiring significant computational effort. Finally, to reduce the error rate in the classification of crystalline and liquid molecules, it has been proposed to combine two parameters *q̅*_*l*_, following the methodology of Desgranges and Delhommelle.^65^ The combination of the lower *q̅*_*l*_ misclassification error values allows a more accurate separation line to be drawn between crystalline and liquid molecules. This reduces the misclassification rate with respect to the individual values. The final expression proposed by Lechner and Dellago^53^ is as follows

3 $${\mathrm{q̅}}_{l}\left(i\right)=\sqrt{\frac{4\pi }{2l+1}\sum_{m=-l}^{l}|{\mathrm{q̅}}_{\mathrm{lm}}\left(i\right){|}^{2}}$$

### Simulation Details

All calculations used in this study to find the best possible combination of parameters correspond to molecular dynamics simulations run using the OPLS-173 AA (Optimized Potentials for Liquid Simulations-All Atom) force field performed in version 2021.5 GROMACS.^66–68^ The two-dimensional graphical representations were plotted using XMGrace software. The images of the systems were acquired using Visual Molecular Dynamics (VMD),^69,70^ with Tachyon ray tracing system.^71^ Regarding the hardware, the simulations were executed using an Intel Xeon Ice Lake 8352 processor with NVIDIA A100-PCIE-40 GB GPU. The hardware used is part of the “Finisterrae III” cluster, whose resources were provided by CESGA (Galician Supercomputing Centre).

For the calculations, the V-rescale thermostat has been used together with an anisotropic Parrinello–Rahman barostat for the molecular dynamics integrator, keeping temperature and pressure constant in NPT simulations, where the dimensions of the simulation box can be varied independently. The time constants for the selected thermostat and barostat have been τ_T_ = 0.1 ps and τ_P_ = 1.0 ps, respectively. Cycles of 30 ns have been carried out with a time step d*t* = 0.001 ps and a cutoff of 1.2 ns. Lorentz–Berthelot combination rules were applied in the determination of cross-interactions. For the calculation of intermolecular interactions, the Particle Mesh Ewald (PME) algorithm was used in order to increase efficiency and processing speed.

The coordinates of the HQ molecules of the β phase have been obtained from experimental X-ray diffraction techniques of a xenon clathrate.^73^ The HQ molecular model used^7^ has been described, tested, and used in previous works,^74^ being the result of the application of the OPLS-AA force field with recalculated point electric charges. The different guests used have been defined according to the models listed in Table 1, having also been used in previous studies.^20,26^ For the case of CH_4_, it is modeled as a single Lennard-Jones sphere. Regarding the model used for CO_2_, it consists of a rigid model of three individual atoms with a partial charge distribution that seeks to mimic the natural quadrupole of the molecule.^17,75,76^

**Table 1:** OPLS Atom Types Used and Force Field Parameters,^72^ Guest to Which They Belong, Mass, σ, ϵ, and Charge (δ) for the Molecules Considered in the Simulations

atom	guest	type^72^	mass/a.m.u.	σ/nm	ϵ/kJ mol^–1^	δ/eV
C	CH_4_	opls_066	16.043	0.373	0.1230	0.000
C	CO_2_	opls_157	12.011	0.350	0.2761	0.820
O	CO_2_	opls_180	15.9994	0.290	0.5858	–0.410

## Results and Discussion

In this section, we present the results of the analysis of the local bond order parameters proposed by Lechner and Dellago for two different phases: crystalline HQ clathrate under zero-occupancy conditions and its respective fluid phase. The calculation has been repeated considering CO_2_ and CH_4_ as guests. The proposed combination of order parameters has been used to characterize the growth or decrease of the number of molecules in a cluster during crystallization or melting processes.

### Analysis of the Optimal Parameter Combination Found for Phase Separation

The combination of optimal Lechner and Dellago averaged bond order parameters has been determined, which provides the most suitable framework for the determination of growth or dissociation of HQ clathrates within complex systems. These allow differentiation between molecules that are part of the crystalline phase and those that are part of the fluid phase. The selection of the most appropriate order parameters was carried out following the labeling criteria detailed by Espinosa et al.^77^ and Sanz et al.^78^

For the calculation of the order parameters, the position of the two oxygen atoms of the HQ molecule are used. The neighbor list for each molecule is determined by excluding the oxygen atom of the same molecule, and each of them is used individually to calculate its number in the solid or liquid phase. Each phase within the configurational system is associated with a specific cloud of points, so those parameters that best define the separation between both clouds will be those that best identify the assignment of each molecule to its respective phase. For further information, it is recommended to consult the previous work of Zerón et al.^57^

Figure 1A,B shows the values of *q̅*_12_ *vs q̅*_8_ for the solid phases corresponding to the β-HQ structure at zero occupancy together with their respective pure fluid phase at 0.1 and 100 MPa, respectively. For both phases, temperatures of 300, 350, and 400 K have been tested. A relationship between *q̅*_12_ and *q̅*_8_ values relative to the solid phase is observed to be inversely proportional to the temperature. This relationship is not appreciable in the liquid phase in the analyzed range. The mislabeling associated with it is <0.001%, which provides an almost negligible theoretical uncertainty when determining the assignment of each molecule to one of the phases. The ideal linear combination of the proposed parameters is represented by a black line. It should be noted that the mislabeling obtained corresponds to the minimum that the method can provide, which implies that the method is not able to detect any overlap between the two clouds.

**Figure 1:**
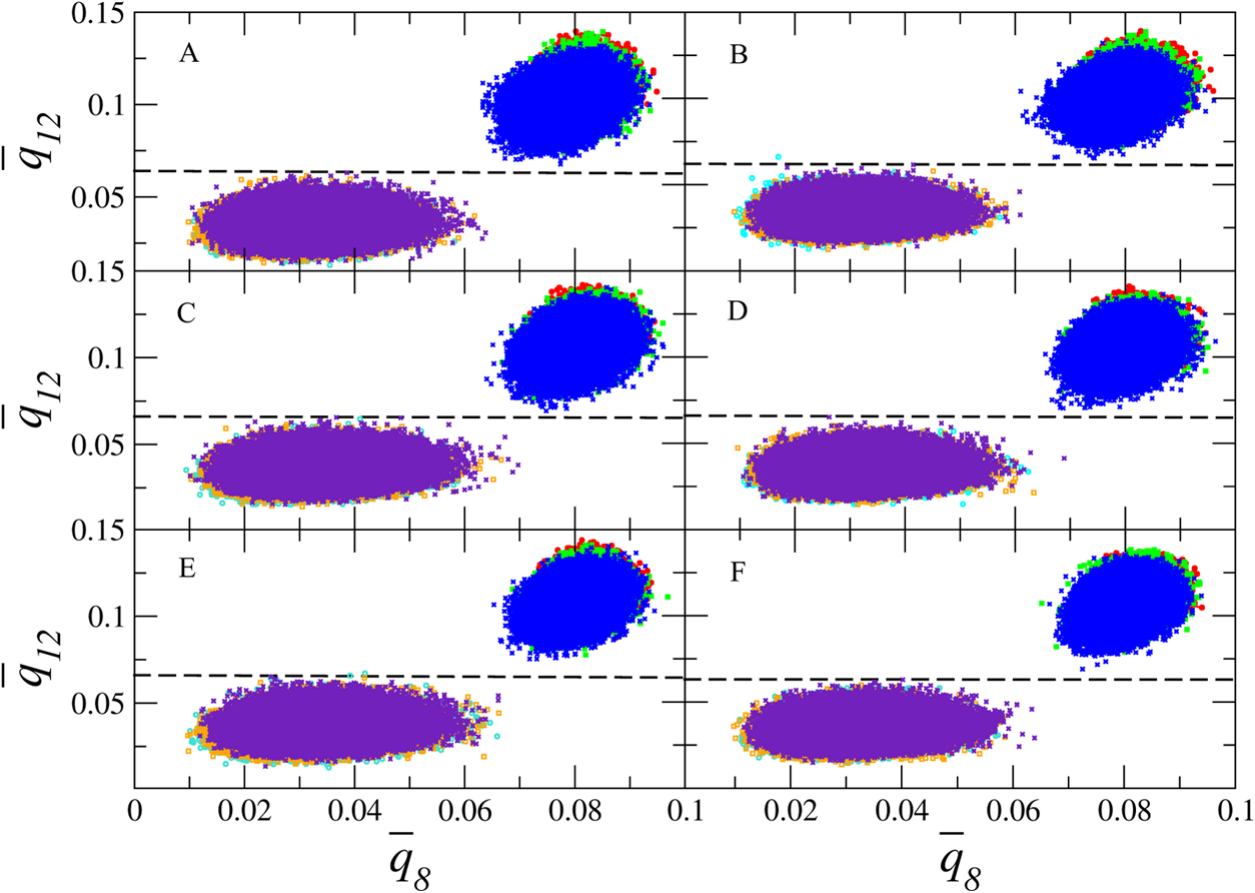
Representation of the values of *q̅*_12_ *vs q̅*_8_ for an empty β-HQ structure (A, B) and with individual occupancy of CO_2_ (C, D) and CH_4_ (E, F) as guest, together with their respective liquid phase at three different temperatures (300, 350, and 400 K) at 0.1 MPa (a) (A, C, E) and 100 MPa (B, D, F). The clouds corresponding to the solid phase are shown by red, green, and blue symbols according to the above-mentioned temperature order. The liquid phase is shown by cyan, orange, and purple symbols, respectively.

The systems corresponding to the guest single occupancy of the β phase compared to the fluid phase containing the guest in solution at a mole fraction *x* ≈ 0.08 are shown in Figure 1C–F, being those relative to CO_2_ at 0.1 and 100 MPa and CH_4_ at the same pressures, respectively. The same thermodynamic conditions of temperature and pressure used in the study of the pure phases have been used for their analysis. Specifically, a system of 288 molecules has been used, the same number that makes up the crystalline seed used, together with 25 guest molecules in solution. In the absence of experimental data on the solubility of CO_2_ in HQ, it is considered that this concentration is sufficiently influential to evaluate the presence of this compound as a solute in the *q̅*_12_–*q̅*_8_ point cloud of liquid HQ. Similarly, there is a shift to higher-order parameter values as the temperature of the system in the solid phase decreases. A complete separation of the clouds corresponding to the crystalline and fluid phases is observed in each case studied, with a mislabeling of less than 0.001%. This indicates that for all the systems proposed in this study in the selected range of conditions, there is a very good ability to discern the membership of a molecule in a cluster.

### Application in Melting and Crystallization Simulations

The proposed parameters have been tested in β-HQ seed growth and fusion processes. A three-phase system, shown in Figure 2, has been considered together with a single-phase system of unoccupied β-HQ clathrate, shown in Figure 3a (side view) and 3b (front view). The former consists of a β-HQ clathrate seed with confined CO_2_ in contact with free guest and fluid-phase HQ, while the latter consists only of the treated crystalline structure under periodic conditions and unoccupied. In both cases, the crystalline phase is composed of 288 HQ molecules. In the case of the three-phase system, it contains 96 guest molecules, corresponding to a ratio of 1:3, one guest particle per cell. The fluid HQ phase of the three-phase system is also composed of 288 molecules, while the free guest phase is composed of 1000 molecules.

**Figure 2:**
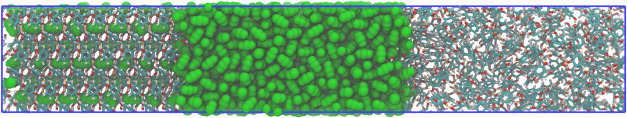
Representation of a three-phase system composed of a β-HQ phase with CO_2_ as guest, free CO_2_, and HQ in a fluid phase. CO_2_ is represented as a molecule composed of three green spheres. In the case of HQ, the skeleton of carbon atoms is shown by light blue segments, while its oxygen and hydrogen atoms are represented by red and white strokes, respectively. The simulation box is represented by a continuous blue line.

**Figure 3:**
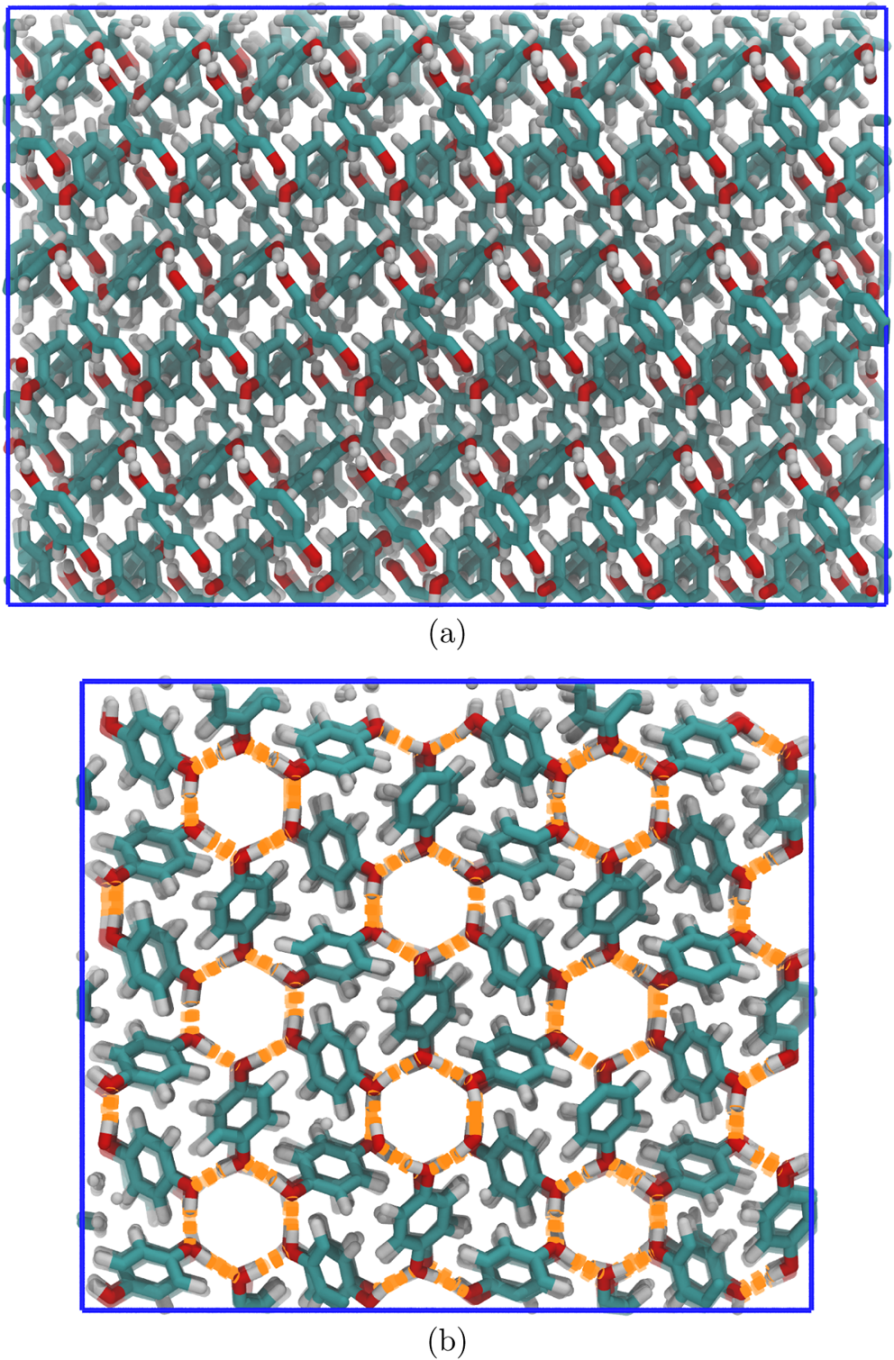
Representation of a β-HQ single-phase system from a side (a) and front (b) viewpoint considering the direction of the channels. In the case of HQ, the carbon atom skeleton is shown by light blue segments, while its oxygen and hydrogen atoms are represented by red and white strokes, respectively. The simulation box is represented by a solid blue line. In the case of the front view, the hydrogen bonds are represented by dashed orange lines as a guide to the eye.

It should be noted that, even if HQ is still present, a clathrate cannot form or grow in the absence of a guest molecule that stabilizes the crystalline structure. This justifies tests of growth in systems consisting of three distinct phases.

Figure 4 shows the representation of the number of molecules belonging to a cluster identified by the combination of parameters noted over the simulation time for a crystal growth process in the three-phase system. This corresponds to the thermodynamic evolution given by the clathrate at 420 K and 100 MPa pressure. A sudden increase in the number of solid-state HQ molecules is observed with time, and a subsequent stabilization that occurs around 15 ns. This trend evidence rigorously the end of crystal growth by depletion of the fluid phase.

**Figure 4:**
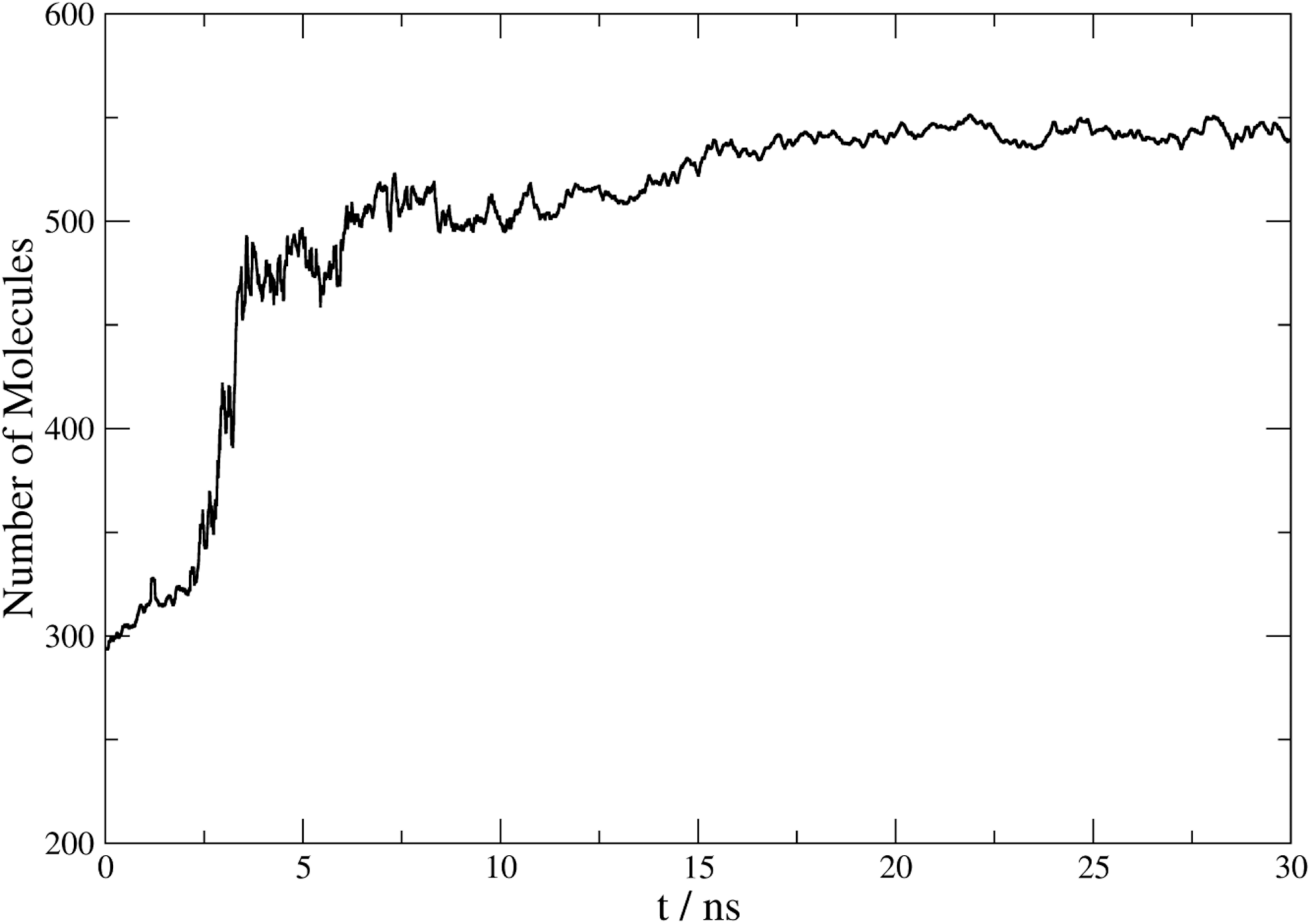
Representation of the number of molecules belonging to a cluster vs time (ns) at 420 K and 100 MPa in an anisotropic regime for a three-phase system of β-HQ, CO_2_, and fluid HQ. Running average was applied with a window of 10 steps.

The proposed parameter combination can quantitatively detect cluster growth. Therefore, it represents a good option to assess the change in a complex HQ system, whose determination is prevented by various circumstances, for example, in a pore confinement situation. It is found that all HQ molecules in the simulated system join the clathrate structure at equilibrium, which is equivalent to a cluster of 576 molecules. This can be verified in Figure 5, which corresponds to a representation of the final state of the system after 30 ns of simulation. This shows that the domain corresponding to the original fluid HQ phase has been organized into a crystalline solid phase corresponding to the β clathrate. At the end of the simulation, the number of HQ molecules remaining exclusively in the fluid phase is estimated to be 38 molecules. The value obtained in this system by the program is 543, with an estimated error close to 1%.

**Figure 5:**
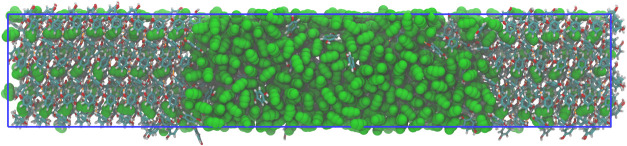
Representation of the three-phase system shown in Figure 2 after reaching equilibrium at 100 MPa and 420 K. CO_2_ is represented as a molecule composed of three green spheres. In the case of HQ, the skeleton of carbon atoms is shown by light blue segments, while its oxygen and hydrogen atoms are represented by red and white strokes, respectively. The simulation box is represented by a solid blue line.

Figure 6 shows the time evolution of the energy of the system. A gradual decrease in energy is observed as the simulation progresses until a plateau is reached at around 17 ns, indicating that an equilibrium state has been reached. This is in coincidence with what can be seen in Figure 4, and a logical correlation can be made between both trends.

**Figure 6:**
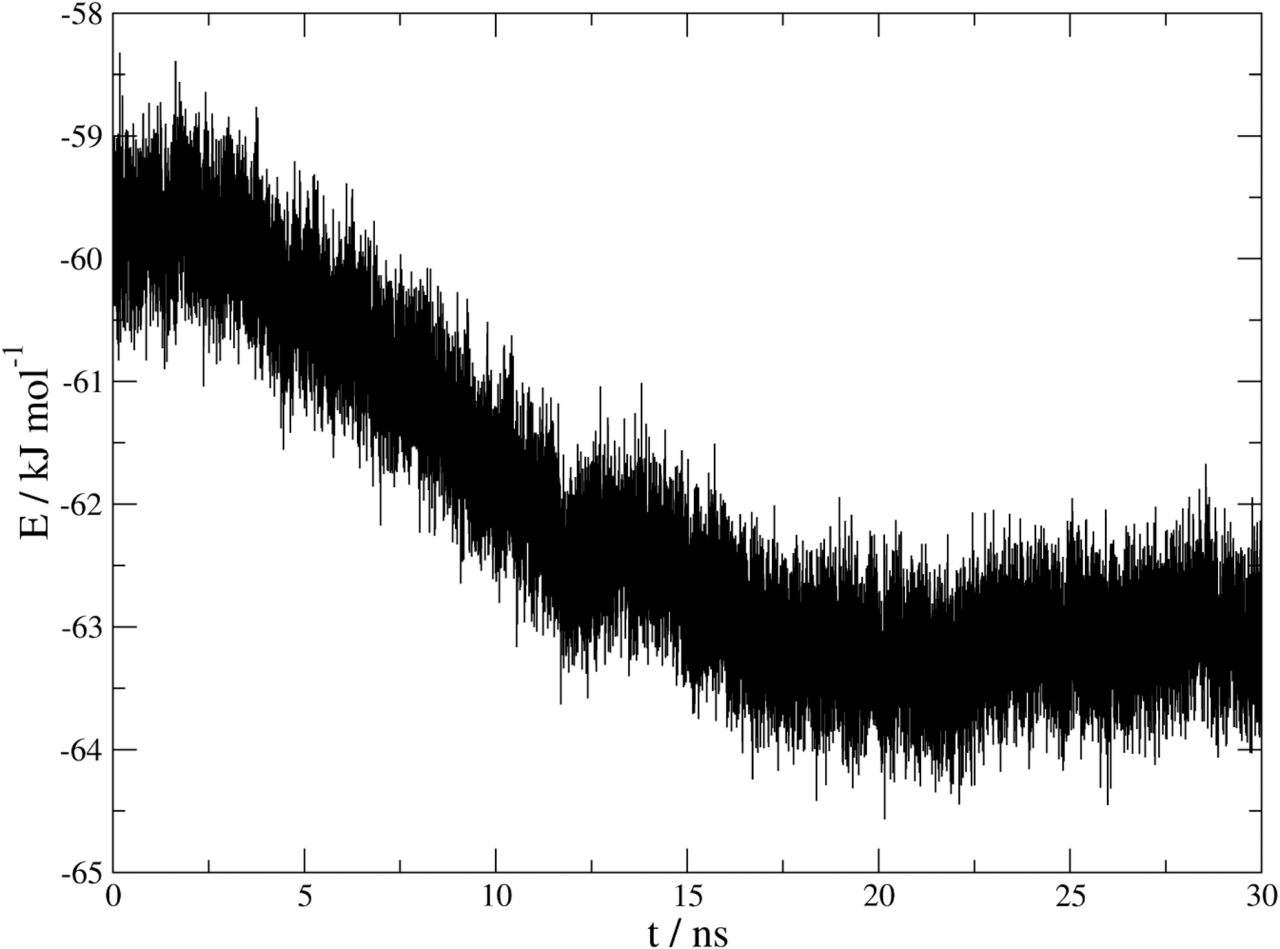
Representation of the energy measured in kJ mol^–1^ at 420 K and 100 MPa in an anisotropic regime of a three-phase system of β-HQ, CO_2_, and fluid HQ.

An example of the application of this method to a dissociation process is shown in Figure 7. This corresponds to the evolution of a single-phase system of β-HQ at 100 MPa pressure and 650 K in an isotropic regime.

**Figure 7:**
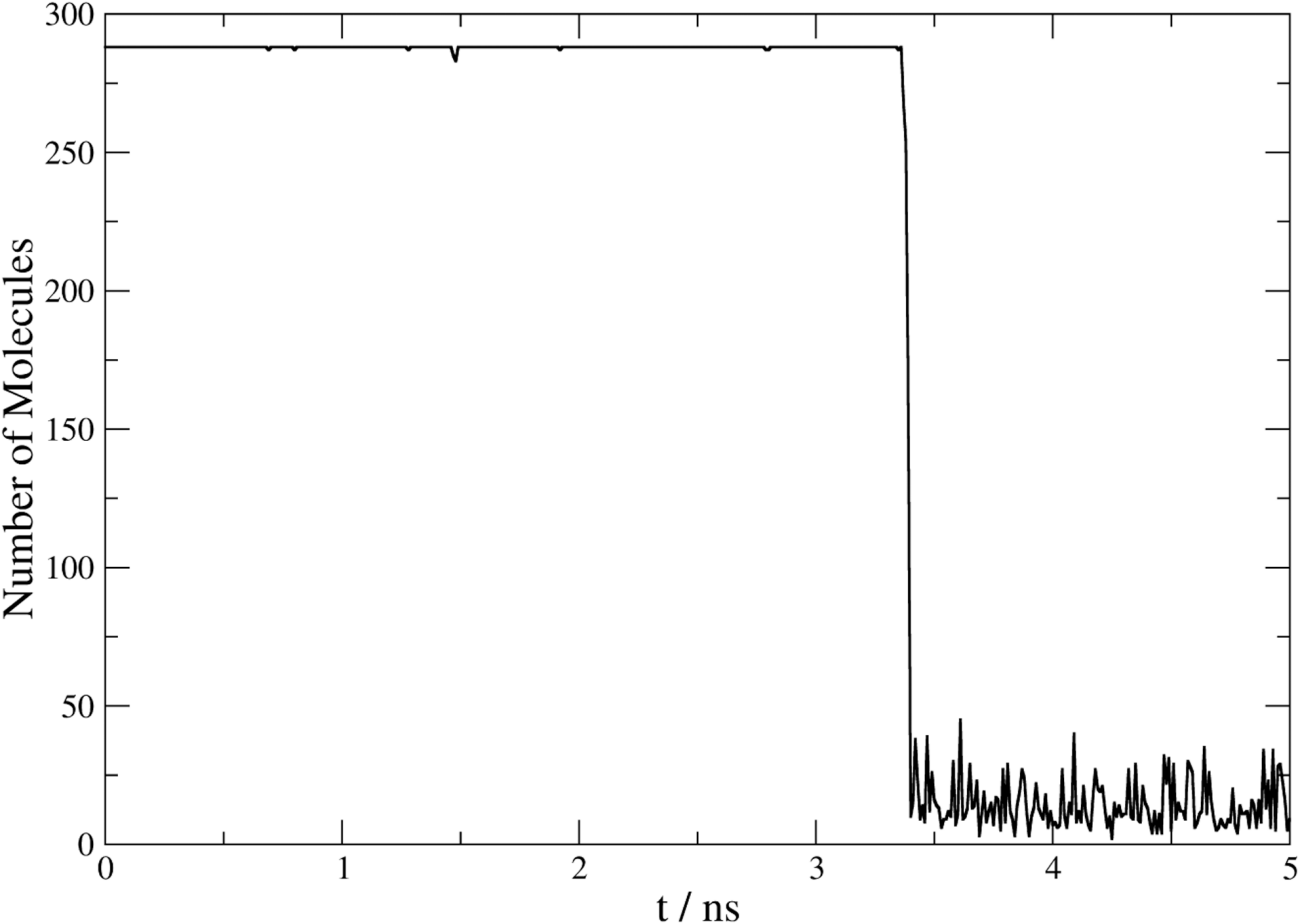
Representation of the number of molecules belonging to a cluster vs time (ns) at 650 K and 100 MPa in an isotropic regime.

A picture of the system once equilibrium has been reached is shown in Figure 8. There is an obvious loss of periodicity due to the dissociation of the crystalline phase. An abrupt decrease in the number of solid molecules is observed near 3.5 ns, which corresponds to the moment when the dissociation occurs. Again, there is a correlation inversely proportional to the energy of the system, shown in Figure 9. There is a sudden increase in energy at 3.5 ns that coincides with the dissociation of the β-HQ cluster detected by the order parameters, indicative of the phase change experienced by the system. Once the crystal has dissociated, the combination of parameters detects clusters between 3 and 34 molecules, this being the noise resulting from occasional clustering of molecules in the fluid phase.

**Figure 8:**
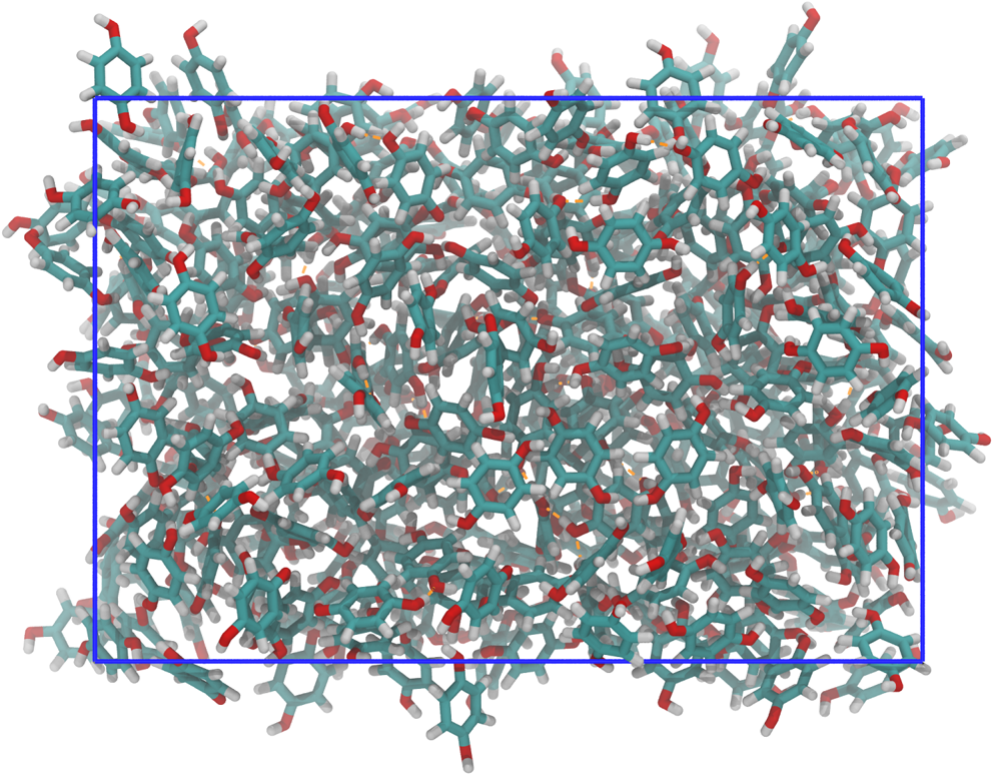
Representation of the single-phase HQ system shown in Figure 3 after reaching equilibrium at 650 K and 100 MPa in an isotropic regime. In the case of HQ, the skeleton of carbon atoms is shown by light blue segments, while its oxygen and hydrogen atoms are represented by red and white strokes, respectively. The simulation box is represented by a solid blue line.

**Figure 9:**
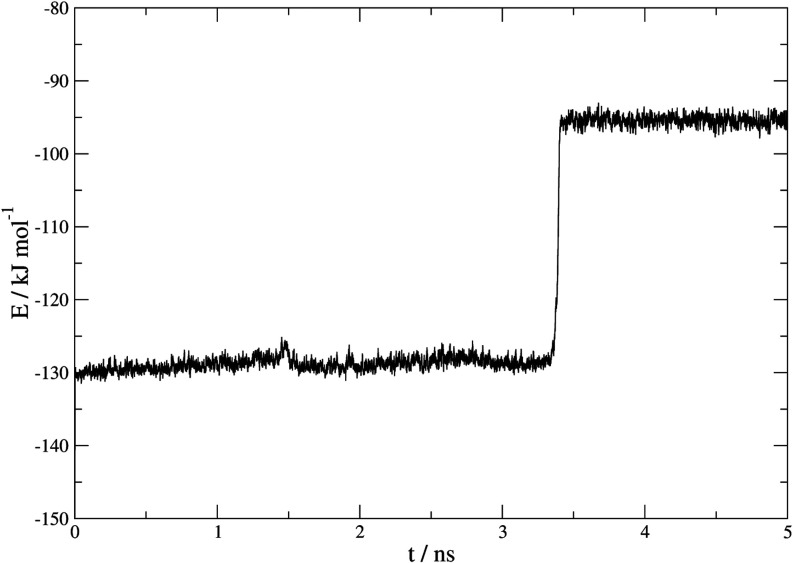
Representation of the energy measured in kJ mol^–1^ of molecules at 650 K and 100 MPa in isotropic regime.

## Conclusions

The analysis of the bond order parameters proposed by Lechner and Dellago^53^ provides a very effective framework for distinguishing between the β clathrate and fluid phases of HQ, with low labeling errors. The optimal combination of these order parameters has been evaluated in order to determine the number of molecules that are part of the crystalline and fluid phases under various conditions. The ability to differentiate these phases is fundamental to understanding phase transitions such as crystallization and melting, and ensures that the method can be applied with confidence to complex systems involving guest molecules such as CO_2_ and CH_4_. The optimal parameter combination found corresponds to that of the order parameters *q̅*_12_–*q̅*_8_, finding an estimated labeling error below 0.001% in every case, considering both a pure system and in the presence of the aforementioned guests.

Using molecular simulation techniques at different temperatures and pressures, it has been observed that the chosen combination of order parameters not only provides a clear separation between the solid and liquid phases, but also works well in capturing the growth dynamics of a seed under periodic boundary conditions. The results show that, during crystallization, the growth of a cluster can be followed over time, with a marked increase in the number of molecules in the solid phase and a stabilization of the energy of the system after the depletion of the liquid phase. These observations underline the power of the method to quantify the thermodynamic evolution of the system, providing valuable insights into the processes driving crystallization. The error in molecule identification, assuming total incorporation into the crystalline phase, was ≈1%.

Furthermore, the application of the bond order parameters to the dissociation processes highlights the versatility of the method, which was able to detect the melting of the clathrate structure as it transitioned to the liquid phase. This phase change was accompanied by a marked increase in energy, which correlated with changes in the number of molecules associated with a cluster. The ability to track such transitions provides a deeper understanding of the thermodynamic behavior of HQ clathrate systems and offers a reliable tool to study phase changes under various environmental conditions.

## Supplementary Material



## Notes

Dissociation process of the β HQ clathrate showing the molecular dynamics simulations with complete dissociation of an originally empty clathrate crystal (Video S1) (MP4)
